# Management of unusual case of self-inflicted penetrating craniocerebral injury by a nail

**DOI:** 10.4103/0974-2700.62115

**Published:** 2010

**Authors:** Kamal Kishore, Sandeep Sahu, Pradeep Bharti, Subhash Dahiya, Ajay Kumar, Anurag Agarwal

**Affiliations:** 1 Department of Anaesthesiology, Sanjay Gandhi Post Graduate Institute of Medical Sciences, Lucknow, U.P., India; 2 Department of Anaesthesiology, Sanjay Gandhi Post Graduate Institute of Medical Sciences, Lucknow, U.P., India; 3 Department of Anaesthesiology, LLRM Medical College, Meerut, U.P., India; 4 Department of Anaesthesiology, LLRM Medical College, Meerut, U.P., India; 5 Department of Anaesthesiology, LLRM Medical College, Meerut, U.P., India; 6 Department of Anaesthesiology, LLRM Medical College, Meerut, U.P., India

**Keywords:** Complications, nail in brain, penetrating craniocerebral injury, suicidal brain injury

## Abstract

During war, sharp high-speed missiles have been driven inside the brain; however, in civilian practice it is rare to see such episodes. An approximately 10-cm long nail was driven inside the brain in an attempt to commit suicide by a schizophrenic patient. The case is being reported for its rarity in civilian practice and as a case of clinical interest. After investigating the patient by plain X-rays and a CT scan, he was operated by a neurosurgical team and the nail was successfully removed. In post-operative phase, patient was given medical and psychiatric care along with psychological counseling. The patient made good uneventful recovery in the post-operative phase.

## INTRODUCTION

Penetrating injury in civilian practice is rare.[1,2] However, cases have been reported by various authors[3,4] of suicidal[5] or homicidal incidences where patients were inflicted injury by means of nails and other objects in order to release evil spirit out of a psychiatric patient or the patient himself nails in a nail to end his life. This is been done by the patients who are suffering from schizophrenia. We are reporting such a case due to its rarity in clinical practice. Till now, only few cases of penetrating brain injury (PBI) by screwdriver have been described,[6–8] in which four cases were homicidal and in two cases accidental. In these six patients, only one patient who got accidental injury survived.

## CASE REPORT

**Figure 1:**
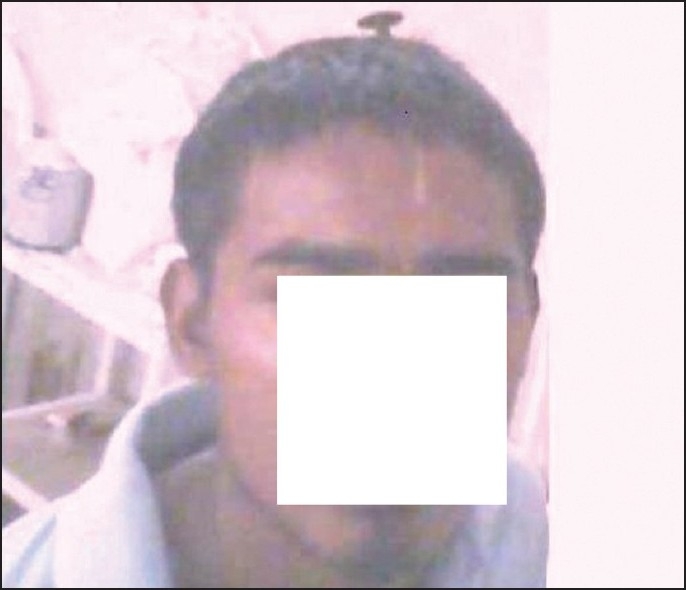
Nail *in situ*

**Figure 2:**
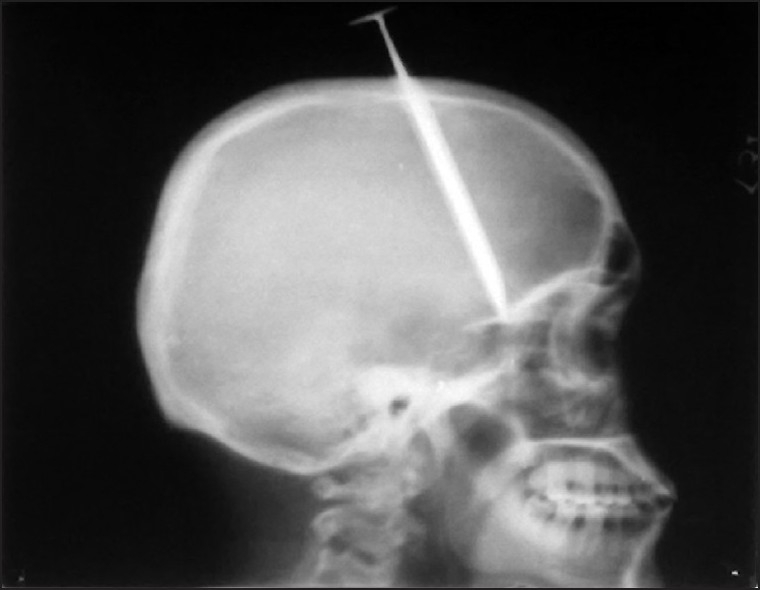
X-ray skull lateral view showing nail depth

**Figure 3:**
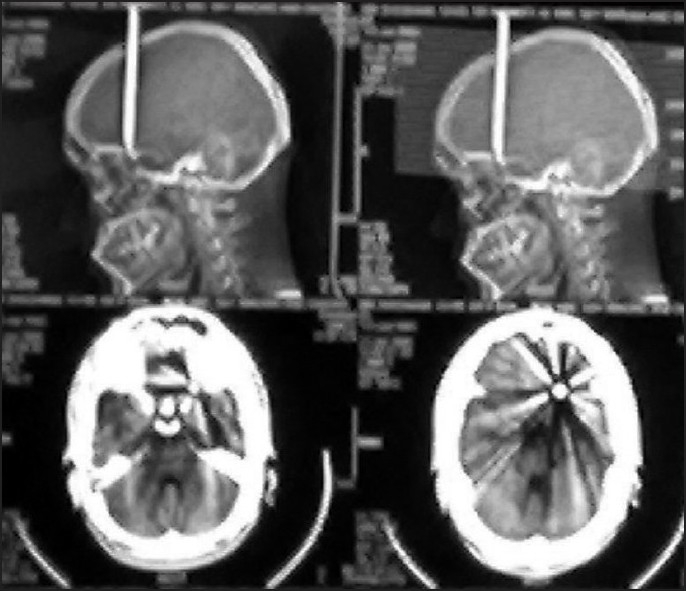
CT scan head-showing craniocerebral injury

**Figure 4:**
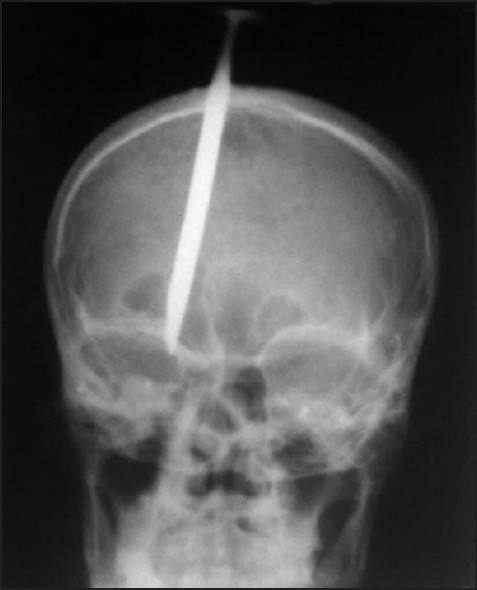
X-ray AP view showing nail *in situ*

This 22-year-old right-handed male, a known case of schizophrenia who had attempted suicide four times earlier, was brought to this hospital as a case of alleged self-inflected injury. As told by the parents, this young man had inflicted self-injury by a nail driven inside his skull with the help of a wooden plank [Figure 1]. It takes about 1.5 h before reaching hospital. At the time of admission in emergency department, on examination this right-handed patient was fully conscious and oriented, talkative with no history of headache, vomiting, seizures, visual disturbances, and with a Glassgow Coma Scale (GCS) of 15\15. There were no focal signs (with intact higher functions, no limbs weakness, sensory loss, bladder bowel involvement, facial asymmetry, and cranial nerves involvement) and a nail was protruding out of his skull in midline approximately 2 cm out near coronal suture. The vitals were as follows: blood pressure 110\66 mmHg, pulse 88\min of normal character, respiratory rate 18\min, pallor +, JVP normal, hydration was adequate, pupil normal size normal reaction, and plantar B\L flexor. This patient had a history of such suicidal attempts four times in the past; last one was almost 4 months back and he was on and off taking anti-psychotic treatment, details of which was not available. Patient was put on conservative treatment along with antibiotics (injection cefurexime BD, amikacin 500 BD, metrogyl 100 ml TDS), antiepileptic drugs (injection eptoin TDS), and antipsychotic treatment sodium valproate, escitalopram, olanzipine, and alprazolam. An urgent plain X-ray of head and neck AP and lateral view [Figures 2 and 3] and non-contract CT scan (NCCT) head [Figure 4] was performed.

Patient was planned for emergency frontal craniotomy. After relevant investigation (hemogram like Hb 12 g%, TLC 8400, DLC P_84_L_14_E_0_M_2_, platelet 2.2 lakhs, PT 11.2\11\,APTT 24\22, serum electrolytes (Na 142,K 4.2), urea 14 mg\dl, creatinine 0.9 mg\dl, blood glucose (R) 140 mg%, and arranging blood products, after written informed consent and proper counseling patient was taken inside the operation theatre. Inj ranitidine 25 mg and inj metoclopramide 10 mg had already been given intramuscularly 45 min earlier, and nasal Ryle's tube was put at the time of admission before operation as prophylaxis for full stomach to prevent aspiration. In operation theatre, invasive arterial and central venous line was put after local anesthetic infiltration to measure beat to beat variability in blood pressure and large volume fluid shift intraoperatively with two large bore intravenous cannula after that craniotomy done under balanced general anesthesia with rapid sequence induction. Intraoperatively colloids, blood products, and inotropes were kept ready with monitoring SPO_2_, ETCO_2_, ECG, urine output, and temperature. Under full aseptic precaution, frontal craniotomy was done. Bone chips were removed around nail without disturbing nail for proper exposure .Then the nail was removed very slowly in line of its original axis to avoid bleeding. Wound site was decontaminated and drain was put, and then aseptically flap was reposited. Vitals were maintained within normal limit during entire intraoperative period. There occurred a small rent in superior sagittal sinus during removal of nail. The bleeding was controlled by the surgeon with the help of gel foams, surgicell, suction, and bipolar cautery. After surgery, the patient was successfully extubated with stable vitals and shifted to intensive care unit. Rest of the postoperative phase was uneventful. Treatment with antibiotic and anti-psychotic drugs was continued similarly to the preoperative phase.

The wound was on the left side of the scalp with the nail directing toward right frontal lobe, which is an eminent center and involved in motor function, memory, language, social, and sexual function. The patient was discharged after 8 days along with psychiatric and psychological counseling. He was conscious and oriented without any neurological deficit.

## DISCUSSION

Penetrating brain injury (PBI) occurs through accidents with firearms and other implements but more often results from intentional injury caused by singular acts of self-destruction or armed conflict in civilian or military arenas. These two latter circumstances differ from each other in significant ways. The principal differences are in the mechanism of injury and outcome (especially death).[9]

Proper perioperative planning by a managing team of emergency physician, neurosurgeon, anesthesiologist, and psychiatrist can save young life of society in such cases. As the patient having nail *in situ* increasing chances of brain infection, we were not in favor to put ICP monitoring device; so we managed the case in the line of central dogma of neurosurgical anesthesia, CPP = MAP – CVP\ICP (whichever is higher), by monitoring invasive mean arterial pressure (MAP) and central venous pressure. We tried to kept cerebral perfusion pressure (CPP) at desired recommended levels. Few studies on intracranial pressure (ICP) in PBI have been performed, and the majority of those studies do not provide ICP-related data. The role of ICP monitoring and its application in PBI have been incompletely studied. In the available literature, intracranial hypertension appears to be common after PBI and, when present, is predictive of less favorable outcome.[10,11] In PBI, physicians should maintain an index of suspicion for the presence of vascular injury, traumatic subarachnoid hemorrhage, and vasospasm. When these are detected, therapeutic measures analogous to those used outside the setting of trauma are indicated. However, outcome data to judge the efficacy of these interventions are limited and do not support recommendations stronger than treatment options.[12,13]

Rare cases of nail being driven inside the brain have been reported in the literature. These nails are driven by the patient himself who is suffering from schizophrenia or severe depression, in an attempt to commit suicide or by the so-called purifier to cure the brain and to let the evil spirit out of the brain.[14–17] Damage of frontal lobe can show spontaneous facial expression and Broca's aphasia[16,17], which were not there in our case. Rare cases of a knife been forced into skull have been reported as a homicidal attempt. Incidence of those cases who have died in such episodes cannot be commented as they are not reported; however, those who have survived and reached the center where neurological care can be provided did survive.[16–18] On the basis of the present body of literature, the question of timing of surgery has not been adequately studied to make evidence-based recommendations. Clearly, the general practice is to operate as soon as the indications for surgery are recognized; if intracranial surgery is indicated, this should also be performed promptly. If the intracranial findings are equivocal, follow-up CT scanning and ICP monitoring should be strongly considered as adjuncts to determine the need for and timing of surgical intervention.[19,20] We followed the same lineage of follow-up at 48 h; apart from that, we got NCCT head with suggested findings of hemorrhagic contusion with postoperative bone defect. It is recommended to do cerebral angiography to rule out vascular injuries in such types of cases, but due to normal neurological assessment in postoperative period and normal NCCT finding and nonavailability of cerebral angiography at our center, the patient could not undergo this investigation. The risk of infection is maximum as these objects are infected and do not become sterile as it is the case in a high-speed missile where the risk of infection remains for a long time; there are also chances of repeated attempts of suicide in these patients. Although there is a paucity of evidence regarding causative agents of infection in PBI, the available data suggest that a wide variety of organisms may act as agents of infection in these patients. This diversity supports the use of a broad-spectrum antibiotic regimen.[21] So we also had given broad-spectrum coverage of antibiotics.

## CONCLUSION

Such rare cases do come in neurosurgical practice. Proper planning for preoperative, intraoperative, and postoperative care can always save these patients who have come alive to the hospital. It is important that these cases be followed for infection. Psychiatric care along with psychological counseling of the patient and the relatives is mandatory to prevent recurrence of such an event in future.
